# Simulation of early DNA damage after the irradiation of a fibroblast cell nucleus using Geant4-DNA

**DOI:** 10.1038/s41598-017-11851-4

**Published:** 2017-09-20

**Authors:** Sylvain Meylan, Sébastien Incerti, Mathieu Karamitros, Nicolas Tang, Marta Bueno, Isabelle Clairand, Carmen Villagrasa

**Affiliations:** 1 IRSN, Institut de Radioprotection et de Sûreté Nucléaire, BP17, 92962 Fontenay-aux-Roses, France; 2 Univ, Bordeaux, CENBG, UMR 5797, F-33170 Gradignan, France; 3 CNRS, IN2P3, CENBG, UMR 5797, F-33170 Gradignan, France; 4 Notre Dame Radiation Laboratory, 102 Radiation Research Building Notre Dame, Indiana, 46556 USA

## Abstract

In order to improve the understanding of the mechanisms involved in the generation of early DNA damage, a new calculation chain based on the Geant4-DNA toolkit was developed. This work presents for the first time the simulation of the physical, physicochemical and chemical stages of early radiation damage at the scale of an entire human genome (fibroblast, male) and using Geant4-DNA models. The DnaFabric software was extended to generate and export this nucleus model to a text file with a specific format that can be read by Geant4 user applications. This calculation chain was used to simulate the irradiation of the nucleus by primary protons of different energies (0,5; 0,7; 0,8; 1; 1,5; 2; 3; 4; 5; 10; 20 MeV) and the results, in terms of DNA double strand breaks, agree with experimental data found in the literature (pulsed field electrophoresis technique). These results show that the simulation is consistent and that its parameters are well balanced. Among the different parameters that can be adjusted, our results demonstrate that the criterion used to select direct strand break appears to have a very significant role on the final number of simulated double strand breaks.

## Introduction

The biological effects of ionising radiation are an active field of interdisciplinary research that aims to improve our understanding of their deleterious nature and our ability to predict them. Improvements might have applications in many fields including medicine, radiation protection and space exploration. Better predictive capabilities would improve the accuracy of radiotherapy and hadron therapy as well as of estimates of their risks. One way of addressing this prediction uses a mechanistic approach to study the chain of physical and chemical events triggered by irradiation within a cell and leading to very early radiation induced effects. Many such studies focus on damage to the DNA molecule, considered highly sensitive to radiation^1–6^.

In this work, we use a mechanistic approach with Monte Carlo simulations and we focus on the damage to DNA induced by radiation. Specifically designed Monte Carlo codes, known as track structure codes^7,8^, must be used to adapt the study of the initial energy deposition of ionising radiation to the DNA scale (only a few nanometers). Geant4-DNA^9–12^ processes are an extension of the Geant4^13^ Monte Carlo code that makes possible the track structure simulations used in this work. Moreover, the simulation must be performed within a geometrical model of the DNA target to be able to compute relevant values, such as DNA double strand breaks (DSBs). This model should be accurate enough to discriminate between the physical and chemical interactions that occur within the sensitive volumes of the DNA.

The DNA geometrical models currently used in this research field range from very simple representations based on cylinders^14,15^ to highly complex, advanced and promising depictions describing the DNA components atomistically^4,16^. Their complexity generally makes it hard to adapt them to the different biological conditions that may influence DNA topology, although the lack of complete knowledge of the organisation of the DNA within a cell nucleus may require this adaptation. That is, although the double helix structure of the DNA has been well described, not yet the case for the higher levels of DNA organisation such as chromatin distribution within the chromosome territories. Furthermore, the organisation of the DNA within a nucleus is also dynamic and changes with the cell cycle and the cell type. DnaFabric software^17^ was therefore developed to facilitate the generation of complex DNA models that can go from a few pairs of nucleotides to whole-genome representations. This software makes it possible to generate, modify, and visualise complex DNA geometries which can also be exported for use in Geant4-DNA calculations.

This work presents for the first time the simulation of the physical, physicochemical and chemical stages of early radiation damage at the scale of an entire human genome (fibroblast, male) and using Geant4-DNA models. This simulation takes the form of a calculation chain that is based on several Geant4-DNA user applications and several analysis programs. In the end, the simulation determines the DNA damage produced by the irradiation. This paper presents the first results obtained with this calculation chain for proton irradiation at different energies and compares them with available experimental data. This comparison makes it possible to set some relevant parameters for the calculation and analysis hypothesis.

## Modelling the DNA within a cell nucleus

### DnaFabric software

DnaFabric is a C++ program to generate, edit, display and export complex DNA geometrical models from the nucleotide scale to the entire DNA content of a cell-nucleus. A previous paper^17^ described an early version of the software and presented a first set of DNA geometrical models. That first version, however, was unable to deal with geometries composed of more than 10^5^ elements; in practice, it could only generate and manipulate a DNA fibre of roughly 18 kbp. Recent improvements enable it to work with a cell nucleus filled with an entire human genome of 6 Gbp (36 ⋅ 10^9^ distinct volumes). The geometry generated can then be exported to a text file (extension “.fab2g4dna”) with a specific format that can be read by Geant4 user applications.

Among the various improvements to DnaFabric is a new module (“Engines”), which allows users to implement a simulation to modify a predefined DNA geometrical model. This module includes tools that can work with the hierarchical organisation of the DnaFabric geometrical models and perform multi-threaded simulations while updating the geometry rendered on the visualisation screen. Furthermore, the hierarchical organisation of the DNA models is now based on a graph structure that can define several memory-light placeholder objects used as references to a single memory-heavy object. This refinement of the hierarchical organisation allows DnaFabric to deal with billions of heavy object instances. The visualisation module was also modified to enable it to render such a huge number of objects. In practice, a level of detail (LOD)^18^ management system was implemented to define several 3D representations for each geometrical object. Thus, an object far from the viewpoint can be displayed as a low-detail representation, while an object close to the view point is, on the contrary, fully detailed.

### DNA model

The DNA model used in this work was built with DnaFabric and its elementary pre-implemented geometrical models: a nucleotide pair, histone protein, nucleosome, linker and 5 voxels filled with hetero-chromatin fibres. This section describes these built-in DNA models only briefly, since most have previously been described^17^.

#### From the nucleotide pair to the chromatin fibre

**Figure 1:**
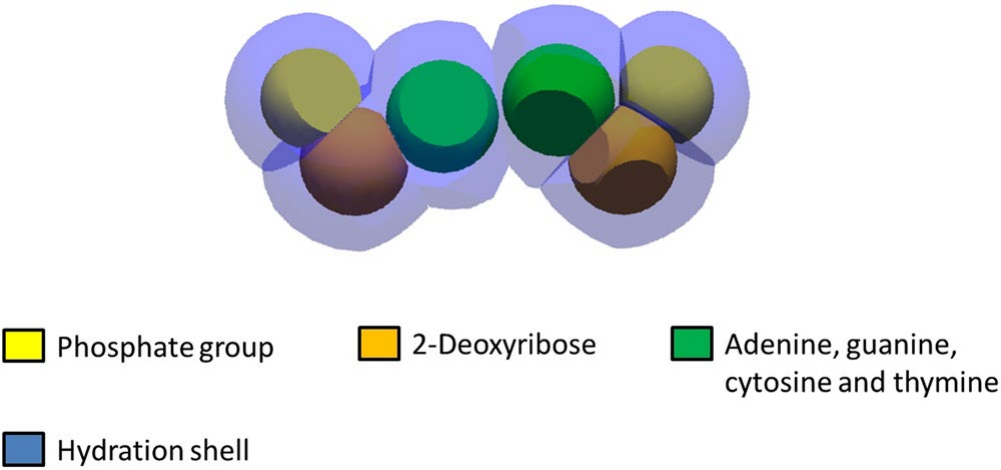
The nucleotide pair model formed by 6 DNA constituents and the surrounding hydration shell (blue)^17^.

Six different spherical volumes were implemented in DnaFabric to represent the DNA constituents used as base units in our model: phosphate, deoxyribose, adenine, guanine, thymine and cytosine. They were used to build nucleotide pairs (the base unit of DNA) such as that presented in Fig. 1. The spherical base units were then cut to ensure that they do not overlap and thus to facilitate the use of the geometry in Geant4-DNA. Additionally, each nucleotide pair was wrapped in a volume representing 24 water molecules^16,17,19^ to model the inner hydration shell of the DNA. Indeed, it is believed that the inner hydration shell that can transfer an ionisation from itself to the DNA^19^. The position and volume of each constituent within the nucleotide pair was calculated from PDB file data provided by the Glactone project^20^. The use of 6 spheres in the nucleotide pair model was chosen instead of an atomistic representation because it speeds-up the computations while not impacting the final outcome since an atomistic level of details is not required in our work.

**Figure 2:**
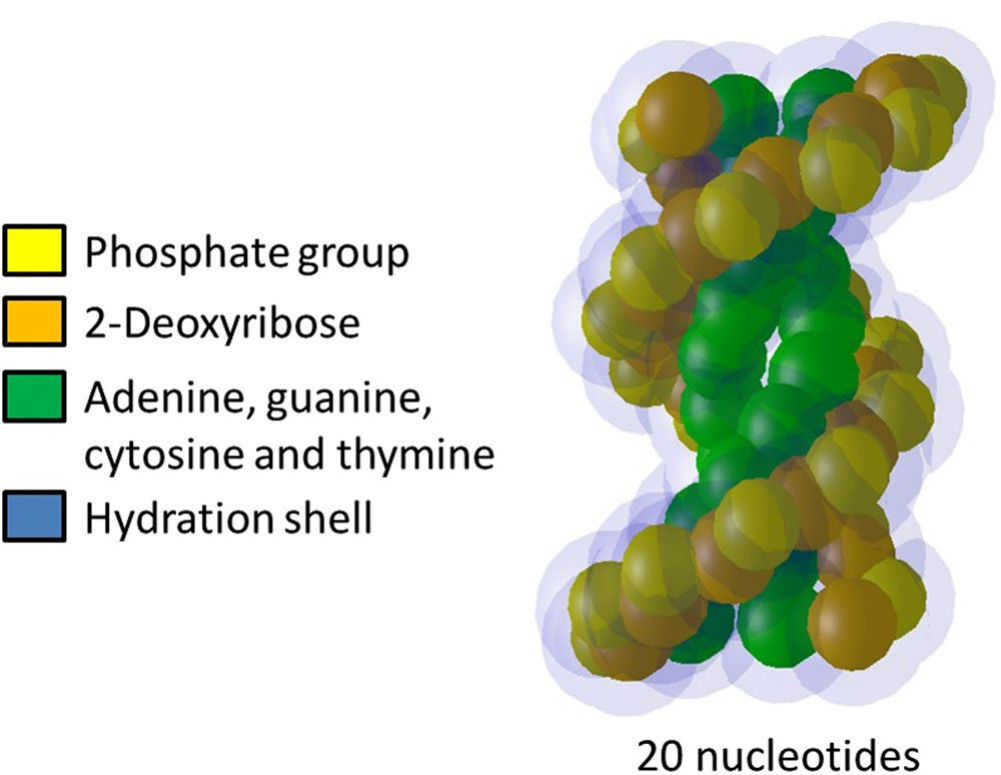
B-DNA double helix built from a stack of 10 pairs of nucleotides.

**Figure 3:**
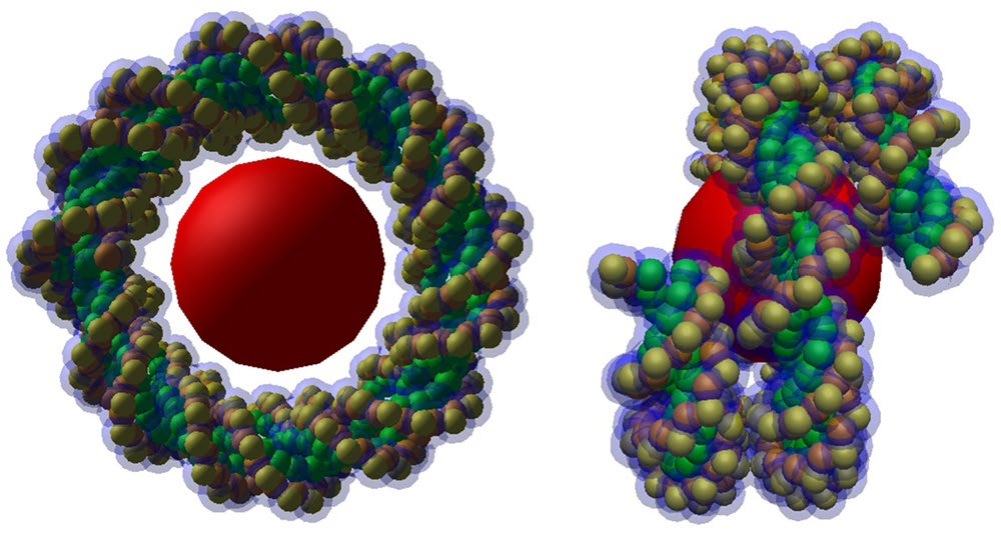
A nucleosome composed of a complex of histone proteins (red sphere) and of a twisted B-DNA double helix.

The B-DNA double helix, which is the most common type of DNA double helix found in living cells^21^ was built with pairs of nucleotides, by stacking several of them along the z axis as described in a previous publication^17^. This produced a B-DNA double helix similar to that depicted in Fig. 2. It was then wisted around a complex of histone proteins, represented by a single red sphere with a radius of 2.4 nm to form a nucleosome, such as that depicted in Fig. 3.

#### Chromatin fibre and voxels

**Figure 4:**
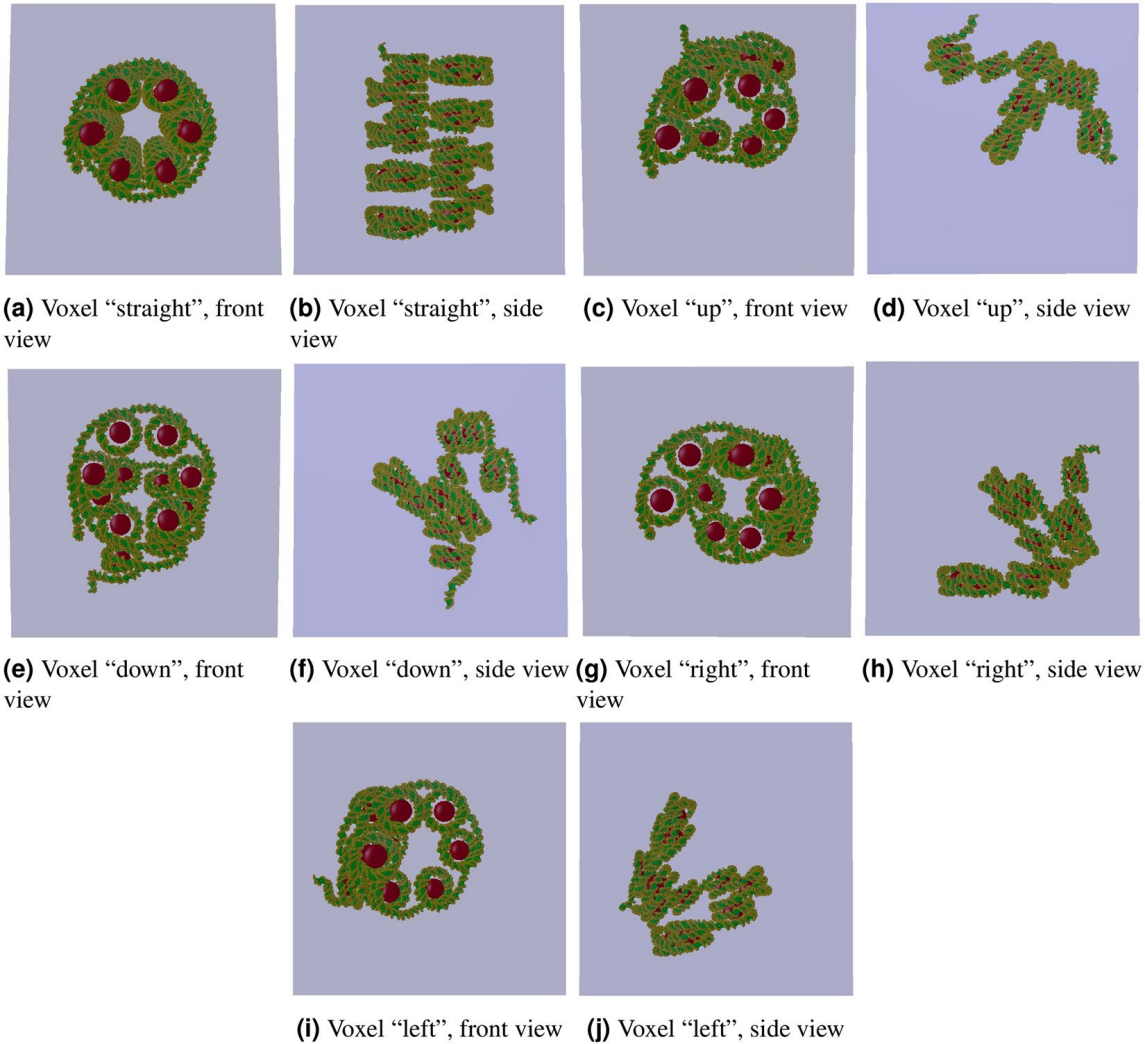
The 5 voxels implemented in DnaFabric: “straight”, “up”, “down”, “right” and “left”.

Several nucleosomes were helically placed and linked together to create a continuous chromatin fibre^17^. In this work, pieces of the fibre (23 nucleosomes) were shaped and oriented to form a set of five different voxel configurations: “straight”, “right”, “left”, “up” and “down” voxels. They are represented in Fig. 4 and their quantitative characteristics summarised in Table 1.

**Table 1:** Characteristics of the 5 voxels: “straight”, “up”, “down”, “right” and “left”.

Characteristics	Voxels				
	Straight	Up	Down	Right	Left
Number of nucleosomes	23	11	11	11	11
Number of nucleotide pairs	4860	2466	2470	2459	2461
Pitch (nm)	10	17	17	17	17
Helix diameter (nm)	10.46	10.46	10.46	10.46	10.46
Number of nucleosomes per turn	6	6	6	6	6
Delta angle (deg)	60	60	60	60	60
Fibre radius (nm)	17	17	17	17	17
Fiber length (nm)	50	39	39	39	39
Voxel length (nm)	50	50	50	50	50
DNA volume (nm^3^)	5.7 ⋅ 10^3^	2.9 ⋅ 10^3^	2.9 ⋅ 10^3^	2.9 ⋅ 10^3^	2.9 ⋅ 10^3^

### Modelling of a fibroblast cell nucleus

Finally, a model of a fibroblast cell nucleus was built and filled with the DNA content of the human male genome. The external shape of the nucleus is ellipsoidal (half-axes dimensions: 9.85, 7.1 and 2.5 *μ*m), and the genome modelled in a hierarchical form: the 5 voxels described above are used to fill chromatin domains. Each domain is represented by a sphere with a radius of 500 nm that contains several hundreds voxels (~10^6^ pairs of nucleotides). Each domain belongs to a human chromosome, which is attributed to a spatial region of the cell nucleus: the chromosome territory. The number of domains to be placed in each chromosome territory is specified in Table 2 and is proportional to the number of base pairs (bp) within each chromosome territory.

**Table 2:** The 23 pairs of chromosomes in the human genome (source: www.ncbi.nlm.nih.gov).

Pairs of chromosomes	Chromosomes	Number of domains
1	1–2	2 × 250
2	3–4	2 × 242
3	5–6	2 × 198
4	7–8	2 × 190
5	9–10	2 × 182
6	11–12	2 × 171
7	13–14	2 × 159
8	15–16	2 × 145
9	17–18	2 × 138
10	19–20	2 × 134
11	21–22	2 × 135
12	23–24	2 × 133
13	25–26	2 × 114
14	27–28	2 × 107
15	29–30	2 × 102
16	31–32	2 × 90
17	33–34	2 × 83
18	35–36	2 × 80
19	37–38	2 × 59
20	39–40	2 × 64
21	41–42	2 × 47
22	43–44	2 × 51
23	X	156
23	Y	57

It should be noted that the process of filling such a cell nucleus model in the G0/G1 phase is itself a three-stage simulation. It requires first the generation of the 46 human chromosomes and their empty domains. DnaFabric does this by randomly positioning one cylinder per chromosome in the cell nucleus. Each cylinder contains all the spherical domains of the chromosome in a “condensed” form. The next stage of the simulation involves “relaxing” the genome in order to obtain a distribution of the domains consistent with the G0/G1 phase. DnaFabric simulates this process according to the model previously described in the literature^22^. Once the relaxed genome is built, the domains are filled with DNA by adding voxels within each domain with a filling algorithm implemented in the “Engines” module. This algorithm generates DNA loops within each domain and ensures that the DNA chromatin fibre is continuous in each chromosome territory.

**Figure 5:**
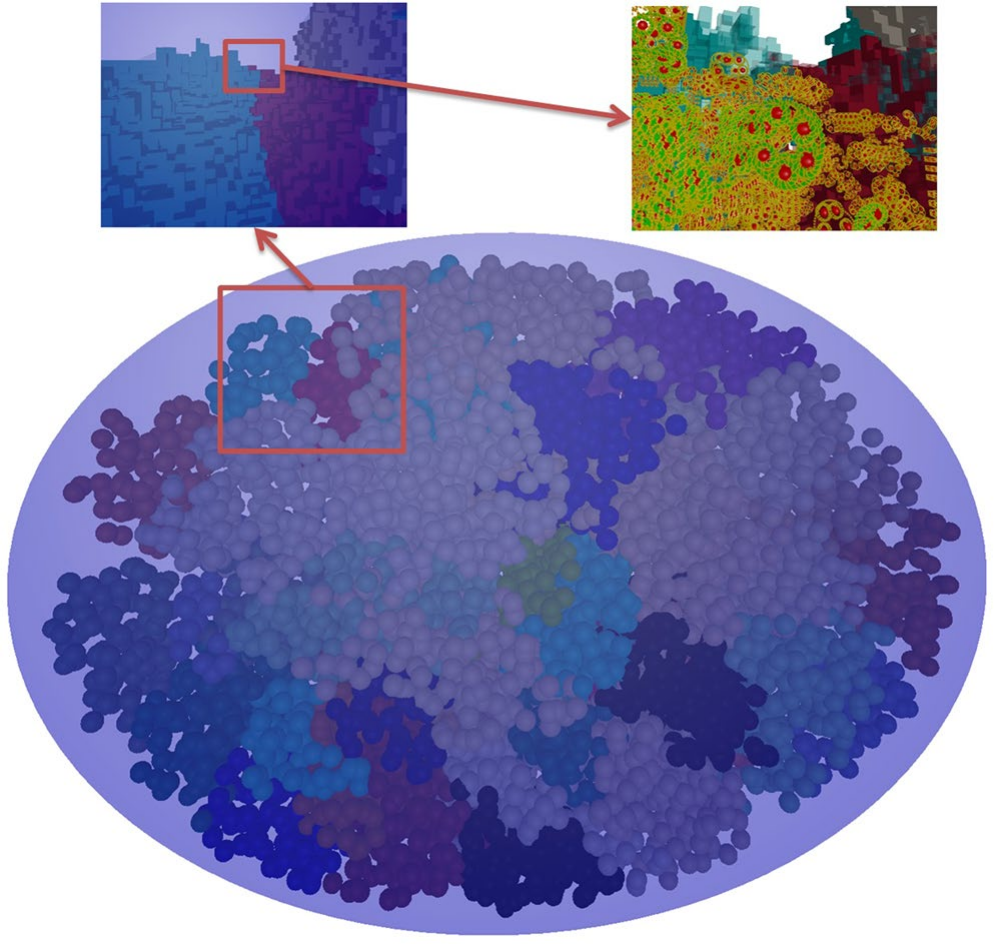
A relaxed and filled fibroblast cell nucleus.The three views presented use three different levels of zoom. The main view (bottom) shows the whole nucleus from a distant point from which only the domains are visible. On the top left, the view is that of a zoom in on the red boxed area of the main view. At this level, it is possible to see the voxels as cubes. On the top right, the view comes from another zoom in on the red boxed area of the top left view. In this view, the detail of the DNA within the voxels is at a molecular level.

Once the filling process is complete, the nucleus model can be exported to a “.fab2g4dna” file for use in the Geant4-DNA simulations. Figure 5 illustrates the fibroblast cell nucleus used in this work and the DNA structure at different scales.

### Modelling the irradiation of a cell nucleus

A calculation chain was developed to simulate the physical, physicochemical and chemical stages triggered by irradiation of a cell nucleus. The modular structure of the chain makes it possible to separate the main stages of the simulation, thus improving the readability of the code and allowing users to run the modules independently.

The calculation chain comprises 7 programs and 7 scripts to be executed in a specific order. The sequencing of these programs is illustrated schematically in Supplementary Figure 1.

The simulation of the physical, physicochemical and chemical stages in this chain uses a slightly modified version of the Geant4.10.1 source code. The user applications contained in the chain can handle the files exported by DnaFabric (“.fab2g4dna”) that enable it to consider the full content of the human genome (6.4 ⋅ 10^9^ nucleotide pairs) during the simulation. The aim of the calculation chain is to compute the yields of DSBs generated per primary particle from both the direct (physical stage) and indirect (chemical stage) effects. Nevertheless, the user can also access intermediate results to, for example, separate the direct from the indirect DNA strand breaks (SBs) or determine the base damage and its location.

The calculation chain can be divided into four parts:

A Geant4-DNA user application to simulate the physical stage (“phys_geo”).

A Geant4-DNA user application to simulate the physico-chemical and chemical stages (“chem_geo”).

A clustering algorithm to reveal DNA cluster damage with a user-specified distance parameter (“DBScan”).

Several analysis routines and scripts to synchronise the elements of the calculation chain and to process the results it generates.

The main hypotheses, assumptions and parameters associated with each of these parts are detailed in the sections below.

### Simulation of the physical interactions within the DNA geometry

The physical interactions between the incident protons (including secondary electrons) and the DNA target were simulated with the physical models present by default in Geant4-DNA (version 10.1) and already detailed in the literature^9,10,12^. The DNA volumes described in section title:dna_models were filled with liquid water, which constitutes an approximation for biological medium, to simulate the physical interactions because the Geant4-DNA models available in version 10.1 use interaction cross sections in liquid water only.

The geometrical description of the DNA used in this user application (“phys_geo”) comes from six “.fab2g4dna” files. The first file describes the cell nucleus and contains the position and type of each of the voxels within it. The other files detail the DNA content of the five voxels introduced in section title:dna_models. The voxels and nucleus in the user application are imported by a parser that generates the corresponding Geant4 geometry. A cutting algorithm is also used to deal with the geometrical overlaps that can appear after conversion into Geant4 geometry. This algorithm is executed once for each of the 5 voxels included in the simulation.

The introduction of a cell nucleus model and its DNA content into the “phys_geo” user application requires the use of specific features. Specifically, the inclusion of several million voxels into the Geant4 simulation necessitates a parametrisation process to keep the amount of memory required by the simulation at a reasonable level (<10 GB). Parameterisation in Geant4.10.1 allows the user to define a volume in memory only once and then to use this definition to represent a large number of identical volumes in the simulation, which reduces the amount of required memory. This method cannot be used, however, if different types of volumes are parameterised in the same area and if the multithreading mode is enabled, as it is in our case to speed up the simulation. We therefore modify Geant4 to enable us to parameterise the 5 different types of voxels and resolve this issue; principally; some variables were made thread-local to avoid data race issues when the multithreading mode is activated. This modification finally allowed the simulation of the physical stage in a cell nucleus filled with around 6.4 ⋅ 10^9^ pairs of nucleotides.

Two datasets are stored in an ntuple generated by the ROOT-CERN library^23^ during this simulation. The first set is used to calculate the DNA damage induced by the physical interactions (direct effects) and the second to generate the input data for the chemical stage (indirect effects). The simulations can thus be separated so that those for the different stages can be run independently when necessary. The first dataset corresponds to the physical interactions located in the DNA volumes: 2-deoxyribose, phosphate, adenine, guanine, thymine, cytosine and their hydration shells. Specifically, the information recorded there concerns the type of interaction (ionisation, elastic etc.), the particles (type and energy) and the DNA molecules involved (name and spatial localisation). The second set of data includes the water molecules that have been ionised or excited and the solvated electrons (physical characteristics and position). Water molecules and solvated electrons are saved only if they are located within a voxel to limit the size of the output file.

### Physicochemical and chemical stages

Like the physical stage, the physicochemical and chemical stages are simulated with a Geant4 user-application (“chem_geo”) built especially to take the geometrical description of the DNA exported from DnaFabric into account. This time, however, the general idea is to consider the DNA model not as a group of Geant4 physical volumes but as a set of spatially ordered molecules that should not diffuse over time. The modifications introduced in the Geant4-DNA chemistry module (version 10.1)^24^ to make this possible are summarized below:

- A number was associated with each DNA molecule to indicate its DNA strand (1 or 2).
- The capability to deal with coefficients of diffusion equal to zero was introduced to specify that each DNA molecule should be treated as a “static” object.
- A new type of reaction was added to prevent one of the reactants from disappearing. It implements the absorption capacity of the histone proteins, which will remain in the simulation regardless of the molecules they may have absorbed.

The DNA molecules presented in Table 3 are thus included in the simulation and a specific parser is included in the “chem_geo” user application. The parser reads the output of the physical stage simulation to introduce unstable water molecules and solvated electrons into this chemical part of the simulation. The parser then processes the “.fab2g4dna” files to generate and place in the appropriate space the DNA molecules to be included in the simulation. The set of reactions shown in Table 4 was added during this work to the default set of chemical reactions of the Geant4-DNA chemistry model (see also Table 5) to allow the DNA molecules to react with the chemical species induced by irradiation. In particular, only the OH^•^ radical was considered able to react with the DNA molecules^25,26^. The latter is an acceptable approximation in this work because the reactions between 2-deoxyribose and e_aq_ or H^•^ are associated with reaction rates that are much lower^27^ than the one associated with the reaction involving 2-deoxyribose and OH^•^.

**Table 3:** Characteristics of the DNA constituents taken into account during the simulation of the chemical stages^17^.

DNA molecule	Radius (nm)
2-deoxyribose	0.29
Phosphate	0.27
Adenine	0.3
Thymine	0.3
Guanine	0.3
Cytosine	0.3

**Table 4:** Reactions added to the default Geant4-DNA chemical module^27–29^. The last reaction simulates the histone protein as an “absorber”: histone absorbs any molecule that directly touches it (no reaction rate is attributed to it).

Reaction	Reaction rate (10^9^ *M* ^−1^ · *s* ^−1^)
2-deoxyribose + *OH* ^•^	2.5
Adenine + *OH* ^•^	6.10
Guanine + *OH* ^•^	9.20
Thymine + *OH* ^•^	6.40
Cytosine + *OH* ^•^	6.10
Histone + molecule → histone_modified_	—

**Table 5:** Default reactions of the Geant4-DNA chemical module (version 10.1)^11^.

Reaction	reaction rate (10^10^ *M* ^−1^ · *s* ^−1^)
${H}^{\bullet }+{{\rm{e}}}_{aq}^{-}+{H}_{2}O\to O{H}^{-}+{H}_{2}$	2.65
*H* ^•^ + *OH* ^•^ → *H*_2_ *O*	1.44
*H* ^•^ + *H* ^•^ → *H*_2_	1.20
*H*_2_ + *OH* ^•^ → *H* ^•^ + *H*_2_ *O*	4.17 ⋅ 10^−3^
${H}_{2}{O}_{2}+{{\rm{e}}}_{aq}^{-}\to O{H}^{-}+O{H}^{\bullet }$	1.41
${H}_{3}{O}^{+}+{{\rm{e}}}_{aq}^{-}\to {H}^{\bullet }+{H}_{2}O$	2.11
*H*_3_ *O* ^+^ + *OH* ^−^ → 2*H*_2_ *O*	14.3
$O{H}^{\bullet }+{{\rm{e}}}_{aq}^{-}\to O{H}^{\bullet }$	2.95
*OH* ^•^ + *OH* ^•^ → *H*_2_ *O*_2_	0.44
${{\rm{e}}}_{aq}^{-}+{{\rm{e}}}_{aq}^{-}+2{H}_{2}O\to 2O{H}^{-}+{H}_{2}$	0.50

The physicochemical and chemical stages are not simulated in the cell nucleus as a whole; instead, reactions are limited to particular voxels. Moreover, reactants can react with one another only if they are produced by the same track (independent track approximation). The separation of the chemical stage simulation within the different voxels is due to the need to minimise memory use and simulation time. Considering the entire nucleus with its human genome simultaneously during the chemical stage would have required loading about 36 ⋅ 10^9^ individual molecules, which exceeds the limitations of not only the chemistry module (Geant4-DNA version 10.1) but also current hardware. Fragmentation of the simulation to isolated voxels allowed us to reduce the amount of memory required drastically. In such a configuration, each voxel contains no more than 10000 individual molecules, which is easily manageable. The drawback of this separation is the need to run numerous different simulations (one simulation per event/voxel pair). On the other hand, it facilitates the distribution of the chemical stage simulations on multiple threads through the use of pseudo-parallelism (one simulation per thread).

The physicochemical stage is simulated with the default “dissociation channels” given in the chemistry module^11^. The dissociation channels describe how an unstable water molecule that has been ionised or excited during the physical stage will decay into chemical species. These chemical species are then randomly placed in a sphere of 1 nm centered on the position of the former unstable water molecule. The resulting chemical species then diffuse and react with each other and with solvated electrons or DNA molecules during the chemical stage. The simulation of the chemical stage takes place in several time steps during which all the molecules move according to their diffusion coefficients^11^. Two potentially reactant molecules can trigger a reaction alongside this movement, initiated either through spatial proximity determined at the end of each time step or during a time step, through the so-called “Brownian Bridge” technique. Scavenging reactions that decrease the number of OH^•^ radicals available to damage the DNA were not specifically modelled in this calculation. A simplification of these scavenging reactions was taken into account by different methods: histone reactions, voxel spatial limitation and, most importantly, by limiting the chemical stage simulation time to 2.5 ns^30^. Other similar simulation codes^31^ use a 10 ns duration but they take into account the scavenging of the chemical species through random absorption of the radicals at each time step.

### Calculation of strand breaks and double strand breaks

#### Determination of direct strand breaks

The data generated during the simulation of the physical stage does not allow direct DNA damage to be identified immediately. More specifically, the cartography of all the interactions that take place within the DNA is available but does not necessarily correspond to direct damage (SB_direct_) to the DNA molecule. Determinations of which interactions leads to an SB or to base damage and of whether the damage occurs in the atom in which the energy was deposited or if a charge transfer occurs, are still the subject of active research. In general, ionisation and excitation occurring within the DNA are considered able to induce DNA damage under some conditions^1^. It is also commonly accepted that ionisation taking place within the DNA hydration shell can lead to direct DNA damage through a charge transfer process^32^ and that a dissociative attachment^33,34^ can create a resonance effect able to alter the DNA structure^35,36^. The latter finding implies that electrons with energies inferior to those required to ionise or excite DNA molecules can still lead to DNA damage. Precise and complete data about the process by which physical interactions causes direct DNA damage remain sparse. Modelling thus requires making assumptions, and in mechanistic simulations, it usually assumes selection based on the amount of energy deposited in sensitive parts of the DNA. The amount of energy and the sensitive volumes change with each simulation code^31,37^.

In this work, the criterion chosen to calculate the number of SB_direct_ from the energy depositions registered during the simulation of the physical stage is a cumulative deposited energy of at least 17.5 eV^38–40^ in the combined phosphate and 2-deoxyribose (hydration shell included) constituents of a nucleotide pair, the region generally known as the “backbone” of the DNA double helix. Nevertheless, a linear probability was also tested to estimate the influence of this selection process on the amount of DNA damage. This probability increases linearly from 0 for a deposited energy less than 5 eV, to 1 when the deposited energy exceeds 37.5 eV^31^.

It should be noted that energy depositions are computed within DNA but with data about liquid water which constitutes an approximation.

#### Determination of indirect strand breaks

During the simulation of the chemical stage, every chemical reaction defined in the code is saved in an output file for later analysis. In the current implementation of the analysis, used for this work, only reactions between OH^•^ and 2-deoxyribose can generate an indirect SB (SB_indirect_). Those reactions, however, are not all necessarily considered indirect SBs. Instead, when such a reaction is detected, a uniform probability of ~40% $(=\frac{2}{5})$ is applied to decide whether it converts into an indirect SB. This probability is applied because the structure of the DNA chain allows only 2 of every 5 reactive sites of the 2-deoxyribose molecule to be reached by the OH^•^^41,42^. It also implies that an average of 11% of all the chemical reactions between OH^•^ and DNA will lead to an indirect SB.

#### Calculation of double-strand breaks and their complexity

After the SB_direct_ and SB_indirect_ are identified and localized within the DNA geometrical model, a clustering algorithm is used to calculate the number of DSBs. In this work, a DSB is defined as a cluster containing at least two SBs separated by less than 10 bp and with at least one SB per strand. This clustering takes place in a merging process that starts by forming initial clusters of SBs separated by less than 10 bp. The clusters are then merged if they share one of their points (that is, one SB). At the end of the merging procedure, the clusters obtained are composed of at least two SBs. This works describes the final number of SBs contained in each cluster as the cluster (or DSB) complexity. It should be noted that this definition of DSB complexity does not include base damage, as the clustering algorithm considers only the SBs. Similarly, clusters formed of two or more SBs that are all located on the same strand are identified as complex single-strand breaks (SSBs) here. Like for the DSBs, their complexity indicates the number of SBs in the cluster. Any isolated SB not belonging to any cluster is considered a simple SSB.

Finally the calculation chain presented here computes the number of DSBs by applying a set of default parameters that can be easily changed by the user. These are:

- A 17.5 eV threshold for the energy deposited in the backbone of a nucleotide to determine an SB_direct_.
- 40% of the chemical reactions between *OH* ^•^ and a sugar (2-deoxyribose and phosphate) give rise to an SB_indirect_.
- A chemical stage duration of 2.5 ns.
- A cluster defines a DSB if it comprises at least two SBs located on opposite strands and separated by less than 10 bp.

#### Analysis required to compare simulated results with experimental data obtained by pulsed field gel electrophoresis

The simulation performed with the calculation chain provides a set of DSBs and SSBs with their associated complexity per simulated primary particle (pp). However, further steps are required to compare the yield of DSB/pp to experimental data obtained with a technique known as pulsed field gel electrophoresis^43–46^. In this case, our simulated results require further processing to take experimental constraints into account:

1. DSBs are detected indirectly since experimental data are basically numbers of DNA fragments resulting from DSBs generated during the irradiation. Knowing that two DSBs are required to create a DNA fragment, it is possible to deduce a number of DSBs from the number of detected fragments. However, the technique has low resolution and some of the smaller fragments are not detected; this artificially decreases the number of experimental DSBs.
2. Experimental results are not given as DSBs per primary particle (proton) but as DSB per Gy and per Gbp (DSB/Gy/Gbp). Furthermore, it should be noted that several Gy are delivered to the cell nucleus in each experimental irradiation and that extrapolation is used to obtain the number of DSB/Gy/Gbp.

To take these two points into account, an additional analysis routine was added to the calculation chain. In this analysis, the position of each simulated DSB in the human genome is used to calculate the fragment size. If the fragment size is lower than the detection threshold of the experimental data (10000 bp), it is removed. A final number of fragments per primary particle (pp) is therefore obtained, which takes into account the resolution constraints of pulsed field gel electrophoresis experiments. This number of fragments corresponds to a number of distant DSBs per primary particle (*DSB*_*distant*_/pp). It is, then, multiplied by the yield of primary particles required to deposit 1 Gy in the cell nucleus and divided by the number of Gbp included in the human genome (diploid cell: ~6.4 Gbp).

### Configuration of the simulations

**Figure 6:**
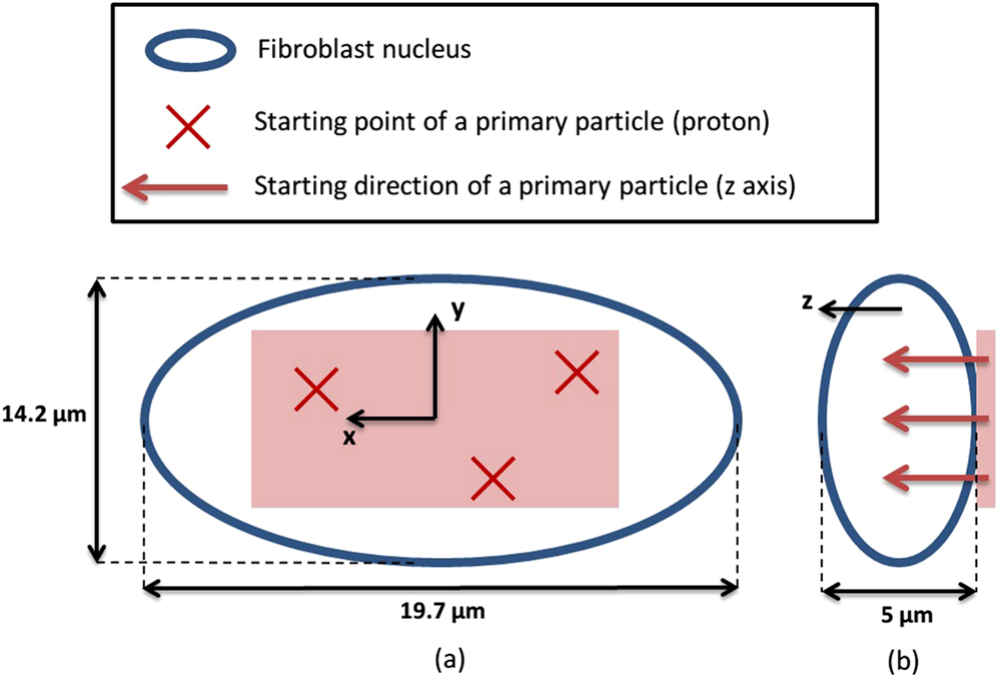
Source of the protons used as primary particles in the simulations. (**a**) Represents a view from the top of the fibroblast nucleus and (**b**) a profile view.

This work simulated the irradiation of a fibroblast cell nucleus. The geometrical model used to represent the fibroblast nucleus was generated as explained in section title:models_cell_nucleus and filled with the DNA content of a human male genome. The primary particles used in these simulations were mono-energetic protons of 0.5 MeV, 0.7 MeV, 0.8 MeV, 1 MeV, 1.5 MeV, 2 MeV, 3 MeV, 4 MeV, 5 MeV, 10 MeV and 20 MeV. LET_d,∞_ associated with these energies in the cell nucleus were calculated (with Geant4) and are shown in Table 6. All secondary electrons were taken into account in the calcultions of the LET_d,∞_ to simulate the electronic equilibrium caused by the broad beam irraditions reported in the literature^43–46^. The primary proton source is represented by a square surface (16 × 12 *μ*m) placed above the cell nucleus. The direction of the particles is parallel to the Z axis. Figure 6 illustrates this configuration. The primary proton source covers only 92% of the volume of the nucleus to avoid the simulation of tracks in the area near the border of the nucleus. Indeed, there is only 3% of the nucleus DNA in these area because the filling algorithm is less effective in such restricted spaces.

**Table 6:** LET_d,∞_ of the protons used in the simulations performed. *d* represents the mean distance travelled within the cell nucleus by each proton and ∞ means that the energy depositions of all the secondary particles are taken into account.

Energy (MeV)	*LET*_*d*,∞_ (keV/*μ*m)
0.5	47.9
0.7	36.5
0.8	32.6
1	27.2
1.5	19.9
2	16.1
3	11.8
4	9.6
5	8.0
10	4.6
20	2.6

The outpout of the simulation is a mean number of DSBs per track that is converted to a DSB yield per Gy and per Gbp using the following normalization:

1 $${N}_{DSB/Gy/Gbp}={N}_{DSB/event}\cdot \frac{{E}_{1Gy}}{\bar{l}\cdot LE{T}_{d,\infty }({E}_{p})\cdot n}\times F$$

with *N*_*DSB*/*Gy*/*Gbp*_(*s*_*bp*_) the number of DSB computed per Gy and Gbp, *N*_*DSB*/*event*_ the number of DSB per track simulated, *E*_1*Gy*_ the amount of deposited energy (eV) required to get one Gy in the nucleus volume, $\bar{l}$ the mean path of each track in the nucleus, *LET*_*d*,∞_(*E*_*p*_) the LET of the primary with an energy of *E*_*p*_, *n* the number of Gbp in the nucleus and *F* a factor to take into account that only 92% of the nucleus volume and 97% of the DNA is irradiated. Specific computations were done to determine the parameters of this equation and the results are:

$$\{\begin{array}{rcl}{E}_{1Gy} & = & 4570\,{\rm{keV}}\\ \bar{l} & = & \mathrm{3,5}\,\mu {\rm{m}}\\ n & = & 6.4\,{\rm{Gbp}}\\ F & = & 0.95\end{array}$$

The statistical relevancy of the simulation results is controled by a dedicated module that starts new batches of 1000 primaries until the statistical uncertainty on the DSB yields is lower than a user specified value. In this work, this value was set to 2% which means that around 5000 primaries were simulated for each energy. Simulations were performed in parallel on a computer cluster which each node was in charge of computing results for one LET value. There were 24 threads per node and the simulations lasted around 3 weeks (depending on the particle energy).

Initially, the default parameters of the calculation chain were used to compute the number of DSBs in the DNA and compare them with both experimental^43–46^ and simulated data^31,40^ from the literature. A minimum fragment size of 10000 bp was used to compute the number of DSB/pp for all the results of section title:dna_damages.

In a second simulation, the criterion used to determine the SB_direct_ (17.5 eV threshold) was modified in a sensitivity analysis to estimate its influence on the final number of DSBs. The threshold was changed to 12.5 and 30 eV and replaced by the linear probability presented above.

## DNA strand breaks simulated with Geant4-DNA

### Comparison of the results with data from the literature

**Figure 7:**
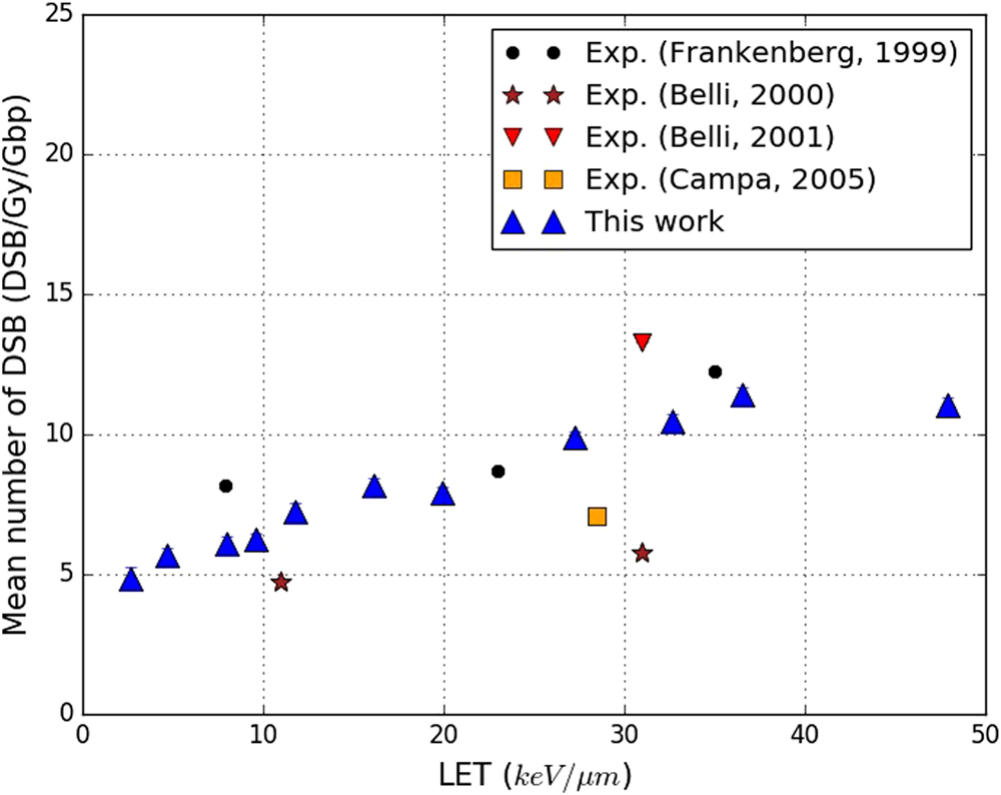
Yields of DSB/Gy/Gbp simulated in this work, when applying the default parameters of the calculation chain (“This work”). The results depend on the LET of the protons used as primary particles. Experimental data from the literature are also presented: Frankenberg *et al*.^43^ (black points), Belli *et al*.^45,46^ (brown stars and red triangles) and Campa *et al*.^44^ (orange squares). Statistical uncertainties are shown for this work unless they are too small to be seen.

Figure 7 shows the yield of DSB/Gy/Gbp simulated in this work compared to experimental data measured by pulsed field gel electrophoresis by: Frankenberg *et al*.^43^, Campa *et al*.^44^ and Belli *et al*.^45,46^. Our simulation reproduced the experimental conditions of Frankenberg *et al*.^43^. The results of Campa *et al*.^44^ and Belli *et al*.^45,46^ came from V-79 Chinese hamster cells, and the lowest fragment size was higher than reported by Frankenberg *et al*.^43^: 23000 bp in the work of Belli *et al*.^45,46^ Despite these differences, the results are included in Fig. 7 to illustrate the scatter of the data. In general, experimental data show that the yield of DSB/Gy/Gbp increases with the LET of the primary protons: the data from Frankenberg *et al*.^43^ start at 8.16 DSB/Gy/Gbp for 7.9 keV/*μ*m and end at 12.22 DSB/Gy/Gbp for 35 keV/*μ*m. The scattering of the data from Belli *et al*.^45,46^ and Campa *et al*.^44^ around those of Frankenberg *et al*.^43^ illustrates the weight of the uncertainties associated with this kind of experimental measurement and the influence of biological factors such as cell type. The results obtained in this work also increase with the LET starting at 5 DSB/Gy/Gbp for 2.6 keV/*μ*m and up to 11.3 DSB/Gy/Gbp for 47.9 keV/*μ*m. Overall, the agreement between our results here and the experimental data is good. This is especially true for the data of Frankenberg *et al*.^43^ around a LET of 20 keV/*μ*m. Interestingly, our results are slightly lower than the experiment for LET lower than 15 keV/*μ*m and higher than 30 keV/*μ*m.

**Figure 8:**
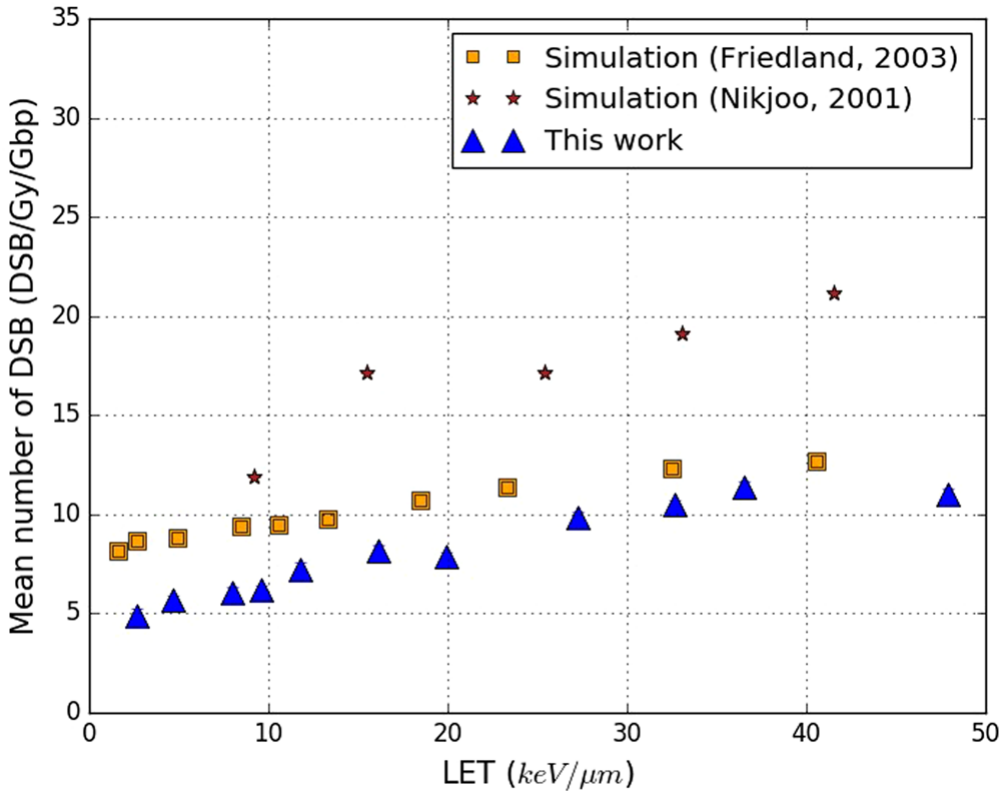
Yields of DSB/Gy/Gbp simulated in this work (blue triangles) when applying the default parameters of the calculation chain (“This work”). Simulated results obtained with the PARTRAC code^31^ (orange squares) and the KURBUC code^40^ (brown stars) are also included. Results are shown as a function of the LET of the primary protons. Statistical uncertainties are shown for this work unless they are too small to be seen.

Figure 8 compares the yield of DSB/Gy/Gbp calculated in this work with results presented by Friedland *et al*.^31^ and Nikjoo *et al*.^40^ simulated respectively with the PARTRAC and KURBUC codes. As previously, our results are based on the default parameters of the calculation chain. It should be noted that some hypotheses differ from those used in PARTRAC or KURBUC. One example is the SB_direct_ selection criterion in PARTRAC^31^, which uses the linear acceptation probability previously described (between 5 and 37.5 eV). The duration of the chemical stage also differs: it is of 10^−9^ s in KURBUC^40^ and 2.5 ⋅ 10^−9^ s in our work. Despite these differences, the results with PARTRAC and KURBUC are comparable to the ones obtained in this work because all of them use similar methodology and the same experimental measurements as references (see Fig. 7). Figure 8 shows that the yield of DSB/Gy/Gbp increases with the LET for all the simulation codes. However, this increase appears to be linear with KURBUC but not with either PARTRAC or our results. In both of the latter cases, the increase of the DSB/Gy/Gbp is less accentuated above a LET value of 35 keV/*μ*m. Furthermore, for LET higher than 35 keV/*μ*m, our results are close to those with PARTRAC (relative difference less than 10%). On the other hand, our results are lower than those with PARTRAC for all LET lower than 35 keV/*μ*m. For example, our results are 35% lower than those with PARTRAC for a LET of 4.6 keV/*μ*m. The results with KURBUC are higher than both those with PARTRAC and our findings. Indeed, the difference between the yields of DSBs computed with KURBUC and in this work varies between 5 and 10 DSB/Gy/Gbp for all LET considered.

**Figure 9:**
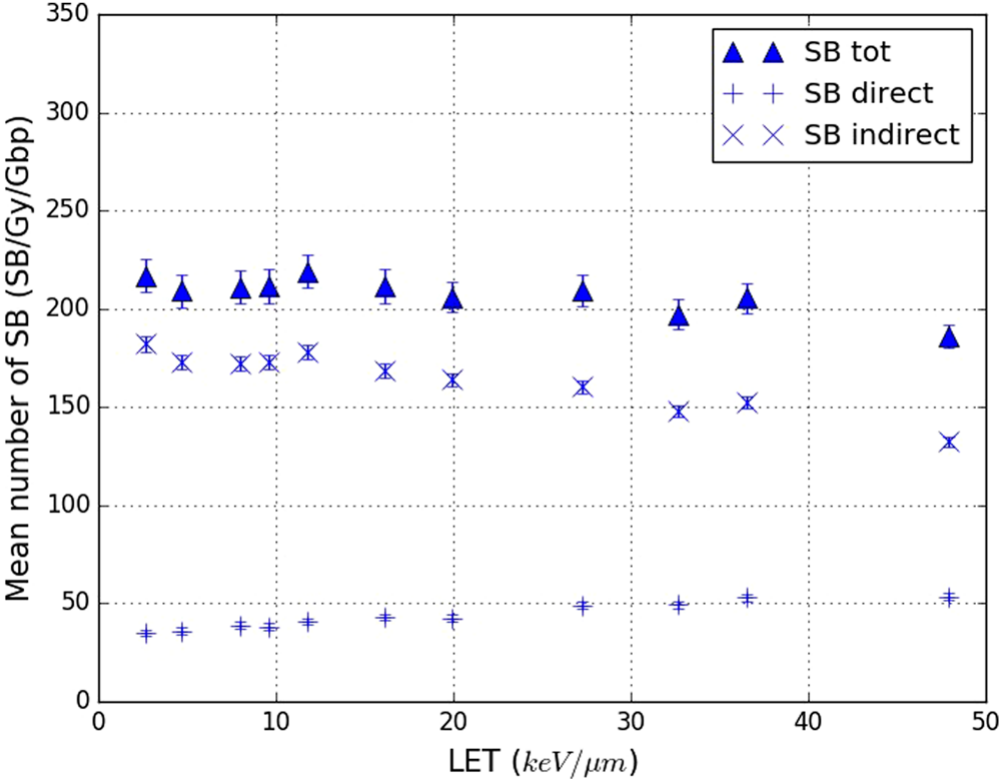
Yields of total, direct and indirect strand breaks (SBs) obtained in this work (default parameters) as a function of the LET of the primary protons. Triangles represents the total number of SBs (SB tot), “+” the SBs from the physical stage (SB direct) and “x” the SBs coming from the chemical stage (SB indirect). Statistical uncertainties are shown unless they are too small to be seen.

The results shown in Figs 7 and 8 above are the yields of DSB/Gy/Gbp calculated by simulating the physical, physicochemical and chemical stages and then processing the SBs produced during the physical and chemical stages to determine these rates. Nonetheless, we can extract from these results the total number of strand breaks (SB_tot_) at the origin of the DSBs and SSBs (cf. section title:sb_calc). We can also discriminate between the SBs from the physical stage (that is, SB_direct_) and from the chemical stage (the SB_indirect_). Figure 9 shows the yield of SB_tot_, SB_direct_ and SB_indirect_ simulated in this work as a function of the LET of the protons used as primary particle. As previously, the results are presented per Gy and per Gbp. The total number of SBs obtained in the simulation (SB_tot_) is almost constant at around 220 for LET values below 20 keV/*μ*m. Nonetheless, for LET values higher than 20 keV/*μ*m, the number of SB_tot_ decreases to 185 for a LET of 47.9 keV/*μ*m. The number of SB_direct_ is quite stable until LET values of 20 keV/*μ*m but substantially lower than those for either SB_tot_ or SB_indirect_. Specifically, there are around 40 SB_direct_ for LET values below 2.6 to 20 keV/*μ*m, equivalent to only 20% of the SB_tot_ and 24% of the SB_indirect_. For LET values higher than 20 keV/*μ*m, the number of SB_direct_ progressively increases to 52 at 47.9 keV/*μ*m.

### Influence of the criterion used to identify direct single-strand breaks

**Figure 10:**
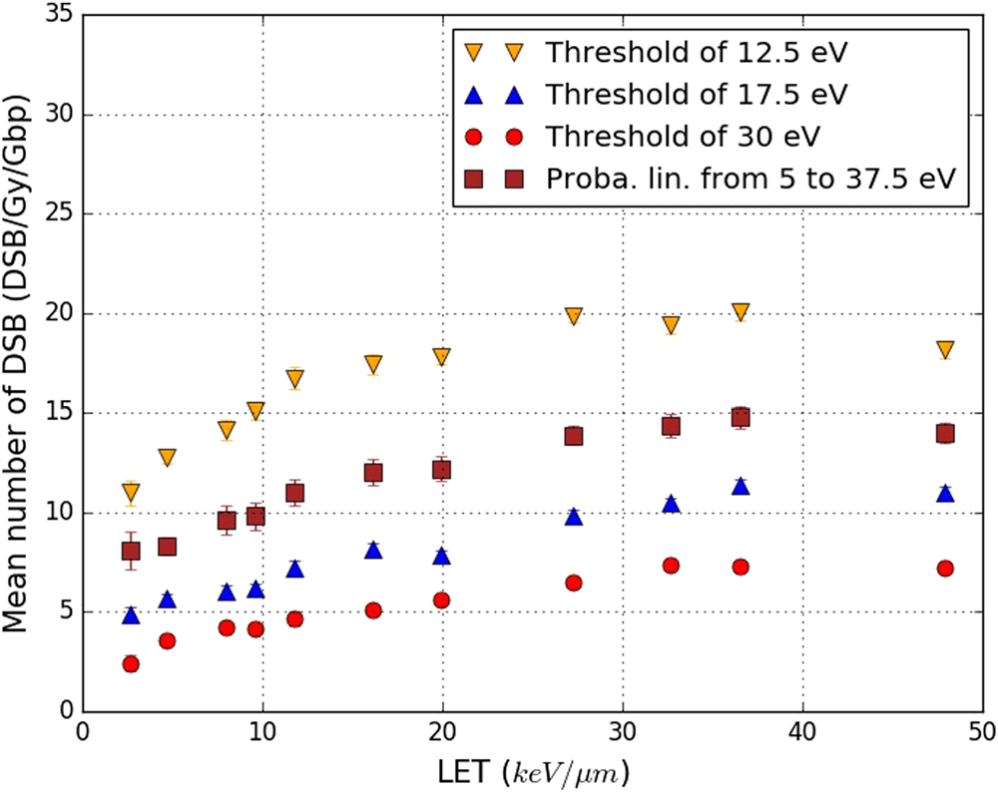
Yields of DSB/Gy/Gbp simulated in this work, as a function of the LET of the primary protons. The calculation chain (“This work”) was used with its default parameters except for the selection criterion used to identify strand breaks produced during the physical stage (SB_direct_). Four sets of results were calculated according to four different selection criteria: threshold of 12.5 eV (yellow triangles), threshold of 17.5 eV (blue triangles, default configuration), threshold of 30 eV (red points) and the linear acceptance probability described at the end of section Configuration of the simulations (red squares). Statistical uncertainties are shown unless they are too small to be seen.

Figure 10 presents four sets of results for the yield of DSB/Gy/Gbp calculated as a function of the LET of the primary protons. They differ in the selection criterion used to identify the SB_direct_ generated during the physical stage of our simulation, as explained in section title:configs. Figure 10 shows that the variations of the yield of DSB/Gy/Gbp with the LET are similar for all four selection criteria. In all cases, the number of DSB/Gy/Gbp increases from 2.6 to 20 keV/*μ*m with an increase factor of roughly 1.7, and then remains almost constant from 20 to 47.9 keV/*μ*m. The use of a threshold of 12.5 eV for the energy deposited in the backbone of a nucleotide yields to the highest number of DSB/Gy/Gbp. The yield of DSB/Gy/Gbp starts at 12 for a LET of 2.6 keV/*μ*m and increases to 18 for a LET of 47.9 keV/*μ*m. The threshold of 17.5 eV (default) reduces the number of DSB/Gy/Gbp by half, it starts at 6 for a LET of 2.6 keV/*μ*m and increases to 11 at a LET of 47.9 keV/*μ*m. A further increase of the deposited energy threshold to 30 eV decreases the simulated numbers of DSB/Gy/Gbp to 3.5 at a LET of 2.6 keV/*μ*m and 7 at 47.9 keV/*μ*m. These three sets of results clearly demonstrate the increasing of the deposited energy threshold (physical stage) decreases the total number of simulated DSB/Gy/Gbp (physical and chemical stages) for all the LET considered in this work. Finally, the use of the linear acceptance probability (see the end of section title:configs) produces a higher number of DSB/Gy/Gbp than the results obtained with the default criterion of our calculation chain (threshold of 17.5 eV) but a lower number than computed with an energy threshold of 12.5 eV.

## Discussion

The comparison between our simulated results, computed in our calculation chain in its default configuration, and the experimental data from the literature (see Fig. 7) shows good agreement between them for the yield of DSB/Gy/Gbp. This agreement is especially good with the data of Frankenberg *et al*.^43^ whose experimental conditions were reproduced in our simulations. Nonetheless, we note the shortage of experimental data available in the literature for proton projectiles with energies between 0 and 20 MeV; moreover, the values for those that do exist are quite scattered. This scattering may be due to the different experimental conditions, in particular the use of different type of cells or different minimum fragment size thresholds, but also from the numerous uncertainties associated with the pulsed field gel electrophoresis technique^43^. Nevertheless, one important consideration is that the general agreement of our simulated results with the experimental data does not mean that the simulation reproduced accurately all the processes involved in the creation of early DNA damage. The default configuration in the calculation chain must use some parameters that are adjusted to keep the simulation balanced in terms of the number of DSBs calculated. In the end, the agreement observed in Fig. 7 shows that the set of default parameters chosen in this work is sufficiently relevant to ensure the consistency of the simulation with the experimental data. Moreover, these parameters are chosen within realistic ranges that can be explained or justified. At the same time, the final values of these parameters and the sensitivity of the final results offer evidence of the importance of the particular mechanism involving them.

The numbers of DSB/Gy/Gbp calculated in this work are similar to those calculated with other simulation codes, as illustrated in Fig. 8. Our results are of the same order of magnitude and vary similarly with the LET of the primary protons. This is especially true for the PARTRAC simulation code which uses an approach very close to that used in this work. It should be noted that KURBUC and PARTRAC also have their own sets of parameters and hypotheses that have been adjusted to ensure their consistency. The differences between these parameters is likely to explain the discrepancies between the yields of simulated DSB/Gy/Gbp. For example, the PARTRAC code uses the linear probability as default selection criterion^31^ while KURBUC and our calculation chain use a threshold of 17.5 eV^40^. Other elements specific to our simulation may also influence the discrepancies: the use of the physical models of the version 10.1 of Geant4, our DNA geometry (more specifically the hydration shell dimensions) and the chemical reactions considered in the simulation.

Figure 9 demonstrates that 80% of the SB, produced by the simulation of the irradiation of a fibroblast cell nucleus were created during the chemical stage which is slightly higher than the ~70% previously reported^31^. The slight increase of the yield of direct SB with the LET was also not reported previously. As for the DSB yields, the parameters and hypothesis specific to our simulation are likely to explain these discrepancies, especially the use of a 17.5 eV threshold. The fact that 80% of the simulated SB were created during the chemical stage raises questions about the real influence of the SB_direct_ selection criterion on the final calculation of the number DSB/Gy/Gbp in our work. That is, the SB_direct_ selection criterion influences only the output of the physical stage, whereas the final number of DSB/Gy/Gbp considers the outputs of both the physical and chemical stages. Figure 10 illustrates the significant impact of this selection criterion on our results. The DSB/Gy/Gbp results depend strongly on the selection criterion chosen, even if their variation with the LET remains similar: an increase phase followed by stabilisation of the number of DSB/Gy/Gbp. This means that the choice of this criterion, even though it can influence only 20% of the total number of SBs simulated (the SB_direct_) can result in very substantial differences in the final number of DSB/Gy/Gbp computed through the simulation of the physical, physicochemical and chemical stages.

**Figure 11:**
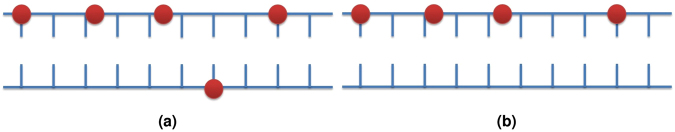
Two clusters [(**a**) and (**b**)] of strand breaks (red points): clusters (**a**) is a double strand-break (DSB) and (**b**) a single-strand break (SSB). Cluster (**a**) contains only one SB on the bottom DNA strand; when this SB is removed cluster (**a**) becomes cluster (**b**).

The significant influence of the number of SB_direct_ on the number of DSB/Gy/Gbp is explained by the DSB determination process (DBScan algorithm), which is conditioned in turn by the creation of a specific type of SB cluster (see section title:ssb_dsb), one that contains at least one SB on each of the two strands of the DNA. Therefore, it is possible for a cluster identified as a DSB to become an SSB if the SB of an opposite strand disappears (see Fig. 11). Modification of the SB_direct_ selection criterion may thus be able to change clusters identified as DSBs into SSBs and to alter substantially the number of simulated DSB/Gy/Gbp.

Note that the simulations performed in this work do not consider the data related to reactions between OH^•^ and DNA bases, although some of these reactions may lead to the appearance of DNA SBs. The processes involved in this conversion are rather complex^47,48^. Overall, theirs contribution to the number of SBs is considered low enough to ignore in this work.

## Conclusion

The calculation chain presented in this work is designed to simulate early DNA damage and is the first simulation tool based on Geant4-DNA that is able to fully simulate the physical, physicochemical and chemical stages of irradiation damage at the scale of a human cell nucleus. Extensions of the DNA models included in DnaFabric software were presented and used to create a fibroblast cell nucleus model filled with the content of the human male genome (diploid cell, $\sim 6.4\cdot {10}^{9}$ pairs of nucleotides). The model was then exported to a file for use in Geant4-DNA simulations. A set of those simulations was created and integrated in the calculation chain, making it possible to simulate the physical, physicochemical and chemical stages that follow the irradiation of a cell nucleus. The simulation of these three stages thus took fully into account the fibroblast cell nucleus model previously generated with its $\sim 6.4\cdot {10}^{9}$ nucleotide pairs.

Simulations were performed to reproduce the irradiation of the fibroblast cell nucleus by primary protons of different energies (0–20 MeV) in order to compute the resulting yields of DSB/Gy/Gbp. The results were then compared with data from experiments that used pulsed field gel electrophoresis^43–46^. Comparison of the simulated and experimental results required inclusion of constraints related to the low resolution of the experiments. This resulted in setting a minimum DNA fragment size of 10000 nucleotide pairs^43^, so that any DNA fragment smaller than that was considered too small to be experimentally detected. Thus, the simulation identified those fragments but ignored them for the calculation of the final number of DSB/Gy/Gbp. In the end, agreement between our results and the experimental data was good and confirmed the coherence of the calculation chain introduced in this work. Our results were also compared with simulated data obtained with other simulation codes^31,40^. The discrepancies observed between our results and those of the other simulation codes illustrate variations that can result from different parameter adjustments and, specifically, different SB_direct_ selection criteria. One selection criterion was shown to influence the number of DSB/Gy/Gbp calculated in our simulation very substantially, although it directly impacts only 20% of all the SBs.

We are currently working at including the reactions of the *e*_*aq*_, hydrogen chemical species and DNA in the simulation. The addition of these reactions together with the use of the data related to the reaction between OH^•^ and DNA bases will enable us to introduce base damage in the simulation. Consideration of base damage is required to extend the simulation from the computation of DSBs to the calculation of chromosome aberrations. Furthermore, the addition of recently published DNA cross sections^49,50^ in the simulation is ongoing work. Their introduction in the simulation will allow to fill the DNA geometry with a composite material which physical properties are closer to DNA than liquid water in terms of interaction probability and amount of energy deposited. The use of these DNA cross sections together with the latest Geant4 physical models for liquid water^51^ will improve the simulation of the physical stage.

The calculation chain created in this work was developed as part of the Geant4 and Geant4-DNA collaborations; the code will be made publicly available in a suitable form for the user community.

## Electronic supplementary material

Supplementeray Figure 1
