# Supplementary material for: Simulation of early DNA damage after the irradiation of a fibroblast cell nucleus using Geant4-DNA
Source: Sci Rep. 2017 Sep 20;7:11923. doi: 10.1038/s41598-017-11851-4 (PMC5607336; doi:10.1038/s41598-017-11851-4)
Supplement: Supplementary file 1 — Supplementeray Figure 1 [file 41598_2017_11851_MOESM1_ESM.pdf]

Simulation of early DNA damage after the irradiation of a fibroblast cell nucleus using Geant4-DNA

Sylvain Meylan[1,*,+], Sébastien Incerti[2,3,+], Mathieu Karamitros[+], Nicolas Tang[1], Marta Bueno[1], Isabelle Clairand[1,+], and Carmen Villagrasa[1,,+]**

[1]IRSN, Institut de Radioprotection et de Sûreté Nucléaire, BP17, 92962 Fontenay-aux-Roses, France
[2]Univ. Bordeaux, CENBG, UMR 5797, F-33170 Gradignan, France
[3]CNRS, IN2P3, CENBG, UMR 5797, F-33170 Gradignan, France
[+]Member of the Geant4-DNA collaboration
[*]sylvain.meylan@symalgo-tech.com
[**]carmen.villagrasa@irsn.fr

ABSTRACT

Supplementary material.

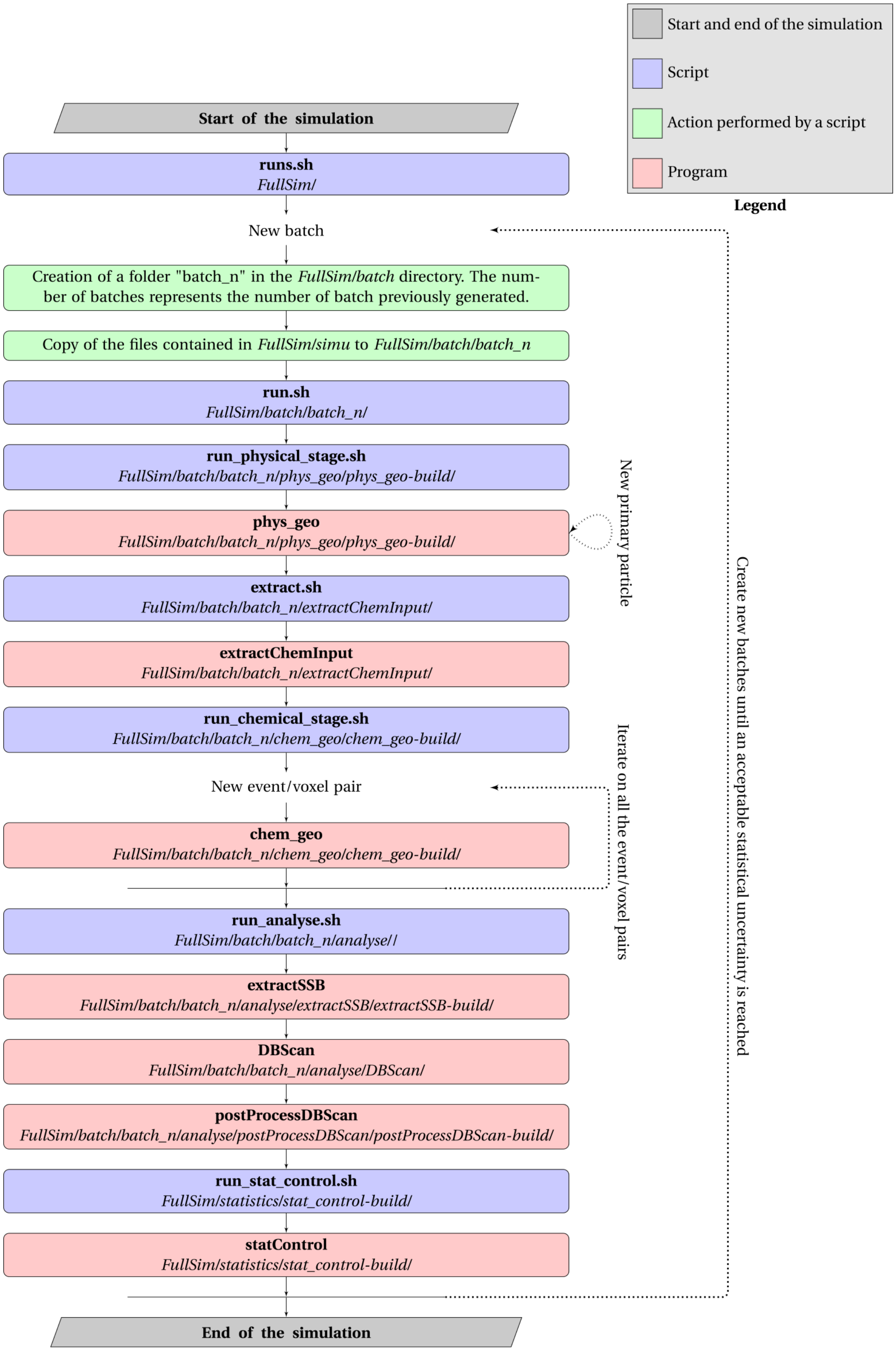

Figure 1. Schematic representation of the calculation chain. Its different programs (red) and scripts (blue) are shown in their order of execution.
